# Functional Alternatives to Alcohol

**DOI:** 10.3390/nu14183761

**Published:** 2022-09-13

**Authors:** David J. Nutt, Robin J. Tyacke, Meg Spriggs, Vanessa Jacoby, Alan D. Borthwick, Delia Belelli

**Affiliations:** GABA Labs (Research) Ltd., Old Town Court, 70 Queensway, Suite 2, Hemel Hempstead HP2 5HD, UK

**Keywords:** ethanol, botanicals, gamma-aminobutyric acid, GABA, GABA_A_R, sociability, alcohol

## Abstract

The consumption of alcohol is associated with well-known health harms and many governments worldwide are actively engaged in devising approaches to reduce them. To this end, a common proposed strategy aims at reducing alcohol consumption. This approach has led to the development of non-alcoholic drinks, which have been especially welcome by younger, wealthier, health-conscious consumers, who have been turning away from alcohol to look toward alternatives. However, a drawback of non-alcoholic drinks is that they do not facilitate social interaction in the way alcohol does, which is the main reason behind social drinking. Therefore, an alternative approach is to develop functional drinks that do not use alcohol yet mimic the positive, pro-social effects of alcohol without the associated harms. This article will discuss (1) current knowledge of how alcohol mediates its effects in the brain, both the desirable, e.g., antistress to facilitate social interactions, and the harmful ones, with a specific focus on the pivotal role played by the gamma-aminobutyric acid (GABA) neurotransmitter system and (2) how this knowledge can be exploited to develop functional safe alternatives to alcohol using either molecules already existing in nature or synthetic ones. This discussion will be complemented by an analysis of the regulatory challenges associated with the novel endeavour of bringing safe, functional alternatives to alcohol from the bench to bars.

## 1. Introduction

**Figure 1:**
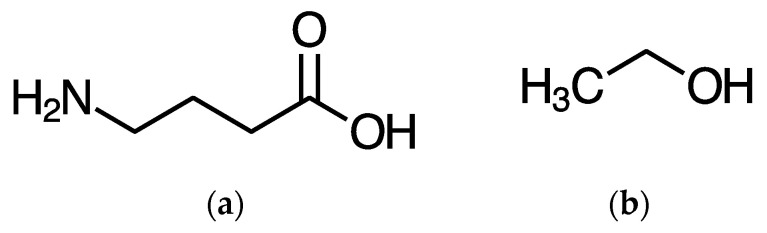
GABA (**a**) and ethanol (alcohol) (**b**) molecules.

The harms of ethanol or ethyl alcohol (with a chemical formula of C_2_H_5_OH, illustrated in Figure 1), colloquially known as alcohol when used recreationally, are well known, with several recent evidence-based assessments from the UK [1], Europe [2], and most recently Australia [3] each putting alcohol at the top of the harms list. Common to all the many strategies to reduce the harms of alcohol, a key element is to reduce consumption [4].

These harms are well-known, and a significant group of people, especially younger, wealthier, health-conscious consumers are turning away from alcohol and looking toward alternatives. This trend has led to perhaps the most interesting development in recent years in regard to alcohol harm reduction—the provision of non-alcoholic drinks that mirror the taste of alcohol. These include zero-alcohol beers and wines and water-based spirit substitutes such as Seedlip, which has juniper (and can have other) flavours that are also found in gin and are used instead of the alcohol spirit, usually with mixers such as tonic water. These zero-alcohol drinks allow drinkers to engage in drinking behaviour without the risks that alcohol intake entails. They also look like alcohol and so can help people who want to avoid questions of why they are not drinking alcohol avoid scrutiny. However, they don’t facilitate social interactions in the way alcohol does, which is why functional drinks that mimic the positive effects of alcohol may have significant advantages: most people who drink socially do so for the pleasurable pro-social effects of alcohol.

The challenge is therefore how to mimic the desired effects of alcohol without using alcohol, and of course ensuring that the alternative is significantly less harmful than the alcohol it seeks to replace [5]. There are two ways to approach this question. The first is to look for other plant-derived intoxicants that have been used by other cultures in place of alcohol, such as cannabis and cava. This approach is fraught with social challenges in many jurisdictions that derive from the illegal status of these drugs. Therefore, the other approach is to find alternatives that mimic the desired effects of alcohol but are free from the undesired harmful ones, an idea first suggested in the UK government Foresight report of 2005 [6]. To do this, one needs to know the mechanisms by which alcohol mediates its desired and undesired effects and then locate molecules in nature, or synthesise ones, that can mimic the former with no—or at least significantly less—impact on the latter. This is the approach we have taken at GABA Labs.

## 2. How Does Alcohol Work?

Alcohol is a small molecule that can interact with a range of different neurotransmitter receptors, many of which have significant roles in brain and bodily function. The full scope of the targets and effects of alcohol across the body and its pharmacokinetics is beyond a detailed review within this article but it is covered in recent excellent review manuscripts [7,8,9,10,11]. In the brain, these interactions appear at different concentrations of alcohol and thus offer the option of in effect targeting a specific neurotransmitter to produce a similar effect to a given concentration of alcohol. The step-wise activity of alcohol on neurotransmitters is beginning to be established by both in vitro and in vivo methods, with some confirmation in human studies.

At low doses, with blood alcohol concentrations of between 0.02% and 0.04%, the relaxing and sociability-promoting effects of alcohol are thought to be due to an enhancement of gamma-aminobutyric acid (GABA) activity in the brain. GABA is the main inhibitory neurotransmitter in the brain, and it is well-established that enhancing GABA function through the specific subtype of GABA_A_ receptors (GABA_A_Rs) calms the brain through decreasing neuronal activity globally. This neuronal calming is thought to explain the relaxing effects of alcohol on the mind as well as on the motor (muscle) systems. The circumstances in which alcohol is usually consumed—generally social events such as bars after work or weddings and funerals—are often accompanied by a degree of social anxiety, especially when strangers are present. Arguably the most powerful and important effect of alcohol in human use is to reduce this social anxiety and so promote sociability and conviviality. There is evidence that human societies have used alcohol for this purpose since the beginning of recorded history and likely before that [12].

As blood levels rise to 0.05–0.08%, the enhancement of GABA continues to increase; this can lead to disinhibition, with louder speech and more hand gesturing, along with some reduced coordination and eye focus. However, other neurotransmitters begin to be activated—dopamine and endorphins. Dopamine is the motive-generating neurotransmitter that is involved in drives, urges, motor activation, and attention. It is likely responsible for the activating and energising effects of alcohol. Dopamine is likely also the neurotransmitter that drives the “more-ish” effects of alcohol (as it does for cocaine) [13]. Thus, dopamine release may underpin the urge to drink more and so result in loss of control over drinking in some people, i.e., bingeing. In addition, adaptive changes in dopamine systems contribute to dependence and craving for alcohol and the low mood seen in withdrawal.

Endorphins are the brain’s internal endogenous opioid system, which dampens down pain and also produces feelings of euphoria. Their release also contributes to the pleasurable effects of alcohol but at the same time can promote loss of control and so lead to bingeing. It seems likely that both dopamine and endorphins contribute to the propensity of some people to become dependent on alcohol. Dopamine is implicated in addiction to other drugs, especially stimulants such as cocaine and methamphetamine, and the endorphin system in addiction to opiates, e.g., heroin [7].

When plasma concentrations rise to over 0.15%, behavioural changes are seen, especially in people with low tolerance. These include unsteadiness, falls, slurred speech, and amnesia (forgetting what you have done). These effects are largely due to the higher concentrations of alcohol blocking glutamate receptors. Glutamate is the main excitatory neurotransmitter in the brain, in effect the “on” switch of the brain. It keeps us awake, is necessary for the laying down of new memories, and keeps vital systems such as breathing going. If glutamate receptors are blocked, we then fall asleep and can go into a state of anaesthesia (ketamine is an anaesthetic that directly blocks glutamate receptors). These are the features that we see in people who have blackouts induced by alcohol, which also blocks glutamate receptors at high concentrations [14]. For an in-depth discussion on the interaction between alcohol and ketamine see [15].

The effects of glutamate blockade are exaggerated by the increased activity of alcohol on GABA receptors, which at these high alcohol concentrations can become irreversible, producing a “double whammy” on consciousness. At some point, the blockade of glutamate excitation stops the respiratory centre of the brain, and so alcohol poisoning leads to death. On the way to this terminal event people may vomit, perhaps a defensive reflex against alcohol poisoning. This is initiated by high concentrations of alcohol stimulating the 5-HT_3_ subtype of serotonin receptors on the vagus nerve, initiating the vomiting reflex [16]. Eventually, chronic alcohol consumption and dependence results in malfunction of the system orchestrating the body response to stressful challenges [17], triggers inflammatory processes, and disrupts the immune response, notably in the brain [18].

## 3. Can Targeting the GABA Receptor Provide Us with an Alcohol Alternative?

In light of this staggered profile of the impacts of alcohol on different neurotransmitter systems, it is possible to consider specifically targeting those that are implicated in the first, and for most people generally the most desired effects, namely relaxation and sociability. Would it then be possible to mimic these desired effects by using molecules that specifically target the GABA system and so are free from the other neurotransmitter interactions that blight many people’s interactions with alcohol? To understand this possibility, we need to briefly explain the complexity and sophistication of GABA in the body and brain.

As already mentioned, in the brain GABA (Figure 1) is the main calming neurotransmitter, working in opposition to the main excitatory neurotransmitter glutamate. GABA is a small molecule with a terminal OH group, similar to alcohol (Figure 1).

GABA has a role in metabolic processes in all living cells, and hence all organisms from bacteria and plants to humans. In addition, it has developed more trophic and communication roles in many organisms and even functions as a signalling messenger amongst different kingdoms of life [19]. Of particular significance is the role of GABA within the syndicate of microorganisms that inhabit the gastrointestinal tract of mammals (and, hence, humans), also known as the microbiome, which has been shown to play an important role in human health including brain function and behaviour [20]. Thus, for example, in the human microbiome GABA is a necessary growth factor for some beneficial bacteria while also being produced by other bacteria [21]. Importantly, *Lactobacillus*, *Lactococcus,* and *Streptococcus Bacteroides* genera and *Bifidobacterium* strains synthesize GABA at concentrations (0.1 to 60.84 mM [22,23]) that affect the function of mammalian and human receptors able to sense and respond to GABA, as described below in this section. Underscoring the importance of bacterial GABA production in the regulation of emotional behaviour, an abundance of GABA-producing *Bacteroides* is associated with less anxiety/distress in healthy women exposed to images with emotional content [24] and in patients with major depressive disorder [21].

In the nervous system, GABA has roles in brain development and neuronal migration, but its main function after birth is as a neurotransmitter that is largely produced in interneurons that are tightly coupled to glutamate pyramidal cells. In this role GABA is released from the interneurons and then acts through binding to proteins called GABA receptors. These come in two types, GABA_A_ receptors (mentioned above), which are ligand-gated negatively charged chloride ion (Cl^−^) channels [25]; and GABA_B_ receptors (GABA_B_Rs), which are G protein-coupled, linked to either voltage-gated K^+^ or Ca^2+^ channels [26,27]. Both receptors are largely inhibitory to the neurons they are located on, and both are affected by alcohol. This review focusses mostly on the GABA_A_Rs, though we mention GABA_B_Rs as a possible alternative target at the end.

There is a large family of GABA_A_Rs that are each made of five protein subunits [25]. At least ten different types of GABA_A_Rs have been identified in mammalian brains, and alcohol acts on many of these. This large number of different receptors provides several advantages. They are expressed in different locations in the brain and on different neuronal types and neuronal cellular domains, providing a fine-grained and sophisticated system of regulatory control. For example, the α1 receptor subtype is highly expressed in the cortex; its stimulation leads to sedation and sleep, the reason why it is the target of most hypnotic drugs, e.g., zolpidem. The *α*2 and *α*3 subtypes are involved in regulating emotions, especially anxiety, and are richly expressed in brain regions modulating emotional states, e.g., the amygdala [28]. The *α*5 has an important role in setting the tonic level of activity and memory in brain areas such as the hippocampus [29].

When a GABA molecule interacts with the GABA_A_R, it opens the associated ion channel so chloride ions can flow across the neuronal membrane into the cell, and for most neurons this inhibits neuronal firing, leading to lower neuronal activity (a calming effect). Alcohols, including ethanol, potentiate the effects of GABA at the GABA_A_R, making the channel opening more efficient, although excessive potentiation of inhibition contributes to the coma-inducing effects of alcohol overdose or poisoning.

## 4. Other Molecules That Work to Enhance GABA

Many other natural and synthetic molecules also interact with the GABA_A_R. Most well-known are those that enhance the effect of GABA—the so-called positive allosteric modulators or PAMs. These include synthetic molecules such as benzodiazepines (BZs) as well as endogenous molecules such as neurosteroids (see Section 5 below). By reducing brain activity, they can stop epileptic seizures and reduce anxiety.

PAM molecules that enhance GABA_A_R function are found in the brain (e.g., neurosteroids) and elsewhere in nature, especially in plants and some fungi. Over the past century a range of synthetic compounds have also been developed and subsequently discovered to share this potentiating action. These include the benzodiazepines, e.g., Valium and Z-drugs that are used to treat anxiety, insomnia, and epileptic seizures. Low doses of some of these have been shown in human studies to have effects that are indistinguishable from low doses of alcohol, suggesting they might be alternatives to alcohol [30].

On the other hand, there are molecules that reduce the effects of GABA (the so-called negative allosteric modulators or NAMs), and these shift the balance between brain inhibition and excitation in favour of the latter, leading to seizures and anxiety. Finally, an important point is that for some of the benzodiazepine PAMs there is a class of molecules that block (antagonise) their positive effect on GABA, e.g., flumazenil. These are clinically available and can very quickly and effectively reverse the sedative actions of these PAMs, e.g., when used for sedation in surgery.

## 5. Neurosteroids and GABA_A_ Receptors

The GABA_A_R receptor is also the target of action of a group of endogenously occurring steroids synthesised in the brain, hence called neurosteroids (NSs) [31]. Amongst neurosteroids that target the GABA_A_R, some metabolites of progesterone, e.g., allopregnanolone or ALLO, devoid of action at classical hormonal receptors, have emerged as potent PAMs of GABA_A_Rs where they bind to a different site to the other PAMs [32,33,34,35]. ALLO’s potent enhancement of GABA_A_Rs has been exploited to therapeutic advantage, and orally active derivates have been developed as anti-epilepsy treatments (ganaxolone [36,37,38] and as antidepressant medicines such as zuranolone [39,40,41]).

In common with BZs, NSs might mimic ethanol as selected NSs can substitute for ethanol in rodents trained with low doses [42], although differences are apparent depending on sex and route of administration [43,44]. The potential for a substitution of alcohol with NSs that is behaviourally beneficial is supported by reports that at least some of the effects of ethanol in rodents appear to be mediated by an increased production of NSs, as described below.

It is well-established that NS production can be halted by blocking an enzyme, 5*α*-reductase, which is crucial for NS synthesis, with a drug known as finasteride [45]. Thus, inhibition of ALLO and ALLO-like NS synthesis with finasteride reduces the hypnotic [46], anxiolytic-like [47], anticonvulsant [48], and antidepressant-like [49] effects of ethanol in rats. In common with studies in rodents, human adolescents admitted to the emergency room due to alcohol intoxication exhibit increased levels of NSs [50,51].

In contrast to the effects of acute administration, chronic alcohol intake results in reduced basal NS levels both in animals and humans. Although species, sex, and regional differences are apparent, these findings potentially indicate that some of the effects associated with chronic alcohol use and discontinuation may be accounted for by the loss of NSs [52].

In summary, current evidence supports a role for GABA_A_R-acting NSs in at least some of the behavioural effects of ethanol, which could be exploited to develop safe alternatives to alcohol. Amongst the GABA_A_R subtypes implicated in the NS-alcohol interaction, *α*2-GABA_A_Rs may be of particular interest as they have been implicated in the modulation of anxiety [29]. Furthermore, mice where the GABA_A_R *α*2 subunit is rendered insensitive to NSs via a single amino acid mutation, i.e., *α*2Q241M KI mice, present an anxiogenic phenotype, which exhibit increased anxiety compared with the corresponding non-mutated and hence GABA_A_R *α*2 NS-sensitive mice [53,54]. These findings both support a role for NSs as endogenous anxiolytics, our own self-made Valium, and reinforce the crucial significance of *α*2-GABA_A_Rs in the modulation of anxiety states.

## 6. The Search for Synthetic Alcohol Alternatives

Molecules that have a degree of selectivity for GABA_A_R subtypes have now been made. As mentioned above, non-selective PAMs such as lorazepam [30] can have alcohol-like effects in humans. PAMs selective for the *α*1 subtype such as zolpidem [55] and zopiclone [56] have been known for decades. They are sedating and used to promote sleep via an action in the cerebral cortex. If used in the daytime, they can have effects similar to alcohol such as dizziness and a “drugged” feeling. They are sometimes used recreationally, which can lead to dependence with withdrawal reactions [57]. Of relevance here is the fact that the reinforcing effects of alcohol are mediated via the *α*1 receptor [58].

There is pre-clinical evidence that GABA PAMs that act on other GABA_A_R subtypes show lower risks of tolerance and abuse, so a number of ligands have been developed as alternative, less-sedating and potentially safer anxiolytics to the non-selective ones such as lorazepam [59]. Interestingly, recent clinical experience suggests one compound selective for the GABA_A_ *α*2 and *α*3 receptor subtype AZD7325 shows some alcohol-like effects, Study 13 within [60]. Other work has suggested that *α*5 receptor subtypes in the hippocampus might also mediate some of the effects of alcohol [61].

Taken together, there is evidence for each of the PAM-sensitive GABA_A_R subtypes mediating possible alcohol-like effects. The question then is which to target and how best to do this? One exciting approach we are using is molecular computational design, made possible by the recent resolution of the crystal structure of mammalian *αβγ* GABA_A_Rs [62,63,64]. The structural basis of ethanol binding to mammalian pentameric ligand-gated ion channels (pLGICs) remains to be fully resolved. Nevertheless, the considerable insight gained into the molecular basis of alcohol modulation of pLGICs that has followed the co-crystallization of the prokaryote receptor GLIC bound to ethanol [65,66] holds the potential to support further computational design of alcohol alternatives.

Of course, we accept that for any novel compound to be sold, it will have to meet the approved criteria for a food or food supplement. This relevant regulatory approval process will involve a significant degree of short- and long-term testing.

However, there are other elements to the search for synthetic alcohol alternatives. We also need to mimic the time course of the effects of alcohol and ensure a much higher safety margin than is the case with ethanol.

A major factor in the pleasurable effects of alcohol is that it works fast—ignoring the mouthfeel and taste (and perhaps a burning throat) with spirits, alcohol effects are noted within 10–15 min, to some extent due to peripheral vasodilation from the alcohol metabolite acetaldehyde. Alcohol’s impact on the brain can be felt within 15–20 min as a sense of relaxation and, in social situations, sociability and integration with others. The time of clearance of alcohol from the body is determined by the dose taken and can be over 12 h from a heavy session, with unwanted enduring impairment as well. We are aiming to markedly reduce the duration of action of our alternative (called Alcarelle at present) to minimise the duration of any impairment. This can be done by designing molecules to be readily and reliably metabolised, with benign metabolites, unlike ethanol. It has long been known that GABA_A_R modulators vary considerably in the duration of action and in many cases this can be explained by differences in their half-life [67]. Although the reasons behind these differences in kinetics are not well understood, they do provide empirical support for the development of new chemical entities with a short half-life.

Safety of any new food product will be paramount. This will of course have to be proven through testing through the approved food safety route. However, there are ways to design compounds that are most likely to meet these criteria. The first requirement is to be highly selective in the targeting of the GABA_A_R-specific subtype and avoid other receptor interactions. In this way, we can avoid the many off-target unwanted and harmful effects of ethanol. The second is to develop PAMs that only partially enhance GABA, so-called partial agonists. This is a well-proven approach to make safer molecules for a range of different neurotransmitter systems. A partial agonist has an intrinsic built-in limitation to have a peak effect less than that of a full agonist: there is a ceiling to its effects that cannot be surpassed by much larger doses. Therefore, the intense intoxication that can occur with excess alcohol can be avoided. The third consideration is to use molecular scaffolds of proven safety. Such an approach allows the risks of idiosyncratic or immune reactions to be minimised. This risk can be further reduced by making very potent molecules so that the total molecular exposure to the body is very low, in the order of milligrams rather than the grams of alcohol used.

Our Alcarelle molecules are designed with all three of these targets at the centre of our research programme. Of course, only when our ingredients have passed through the food-safety testing protocols will we be able to tell if our plans have proved successful. This process is now starting with our lead molecule and should be concluded within a year or so.

## 7. Botanical Alternatives to Alcohol

While synthetic molecules offer probably the best way to develop precision pharmacological agents, the potential for naturally occurring GABA-acting molecules must also be considered. The billion-year-old evolutionary history of GABA from the first monocellular organisms through plants and animal species reveals it to have multiple roles, often relating to stress reduction, just as it does in vertebrates [68]. The long history of GABA as a vital part of biochemical machinery suggests a causal relationship between the foundational role and proliferation of GABA as a stress response mechanism in plant biochemical systems [69] and the seemingly parallel evolution of GABA in vertebrates [70].

It follows that there may be a wide existence of food plants making molecules that act on the GABA system in humans, and indeed this appears to be the case (for review see [71]). This triggered our search for naturally occurring botanic compounds within the human food chain with such activity.

The search was approached from several directions. First, we explored the available literature for plants that were already approved as foodstuffs or as food supplements, and which were known to have been used traditionally in European and other herbal drinks to produce feelings of relaxation and other alcohol-like experiences. From these, we tested the plants that had evidence of GABA activity and began an experimental programme to test different combinations for effects.

Second, we identified a number of molecules thought to be responsible for the GABAergic effect within specific plants. This led to a search of the literature for the known existence of this chemistry in other plants and to testing of the plants identified. This approach has proven to be slow and laborious, largely due to the large amount of data that must be manually investigated. It is possible that this process could be accelerated using AI to identify plants containing GABA-acting molecules. We plan to develop a training set from the plants we have already identified to develop algorithms for predicting candidate molecules in large food-plant molecular databases, when future funding permits.

The level of GABA efficacy for the plant extracts tested was found to be low at the maximum daily dose level permitted under UK food regulations. A final stage in the discovery process was to establish ways to amplify and accelerate the effect of the GABA-acting materials. These include the use of established bioavailability enhancers such as piperine (from black pepper) [72] and other plant extracts that facilitate transport of small molecules across the blood-brain barrier (BBB), e.g., borneol [73].

A range of blends were designed, each incorporating one or more core GABAergic ingredients, one or more ingredients to assist bioavailability, and at least one to assist with crossing the BBB. All have been tested at lab scale for efficacy and positive results obtained for a number of distinct blends. Lead candidate blends were then subjected to taste and texture development and from these a preferred blend was selected for testing. Results indicated strong potential for consumer acceptance. The blend has now been the subject of extensive further development into a premium liquid now known as Sentia Red. This is a vermouth-like drink that can be taken neat or with mixers, e.g., tonic. The herbs selected for Sentia are listed below in descending order of the amount present in the liquid. All have been used for beneficial effects over many centuries, and so are recognized as foods or food supplements: blackberry juice, aronia, magnolia, linden, passionflower, liquorice, ashwagandha, hawthorn, damiana, rose, tulsi, hibiscus, and spice/pepper extracts.

Sentia Red was launched on the UK market as a GABA spirit in January 2021 and has had favourable feedback from the food media and drinkers [74,75,76]. Other differently flavoured versions of Sentia are now being developed, with one planned to be launched this year. In addition, we have begun a programme of studies with several UK universities to evaluate the short- and long-term psychological, physiological, and biochemical impacts of our drinks to rule out the (unlikely) occurrence of undesirable effects from the use of the approved ingredients.

Consumer demand for alcohol-free functional beverage products is growing rapidly. The market is currently worth tens of millions of dollars per year and a wide range of alcohol-free beverage products has become available to consumers.

Other functional alcohol alternative products also target GABA. One beverage in the USA, Kin [77], originally contained phenibut [78], an old Russian anti-anxiety medicine that acts on GABA_B_Rs and gamma hydroxybutyrate receptors ( GHBRs). This has since been removed to comply with US Food and Drug Administration (FDA) regulations [79]. However, one of the Kin drinks also contains GABA itself. Although it is not proven that GABA can enter the brain across the blood-brain barrier, ingested GABA might produce psychological effects through acting on nerves in the gut and/or on the microbiome [80].

Three Spirits is a UK-based company that makes several functional drinks as alternatives to alcohol. Their website [81] states their drinks include herbs that enhance GABA in the brain such as passionflower [82] and lemon balm [83]. Interestingly, they also contain myrcene, which though not GABA-acting does have anti-stress effects [75].

### NS and NS-like Molecules in Plants: An Alcohol Alternative?

NSs such as ALLO are not present in plants but, intriguingly, members of the *Solanaceae* family, which include both food (e.g., tomatoes, potatoes, peppers, and aubergines) and medicinal plants, express enzymes similar to those crucial for the synthesis of NS, namely two isoforms of the rate-limiting enzyme for NS synthesis, 5*α*-reductase, mentioned above [84,85]. Moreover, the existence of 5*α*-reductase activity appears to be a general feature of the plant kingdom, and plant equivalents of the mammalian 5*α*-reductase possess high affinity for and hence can utilise progesterone as a substrate for the synthesis of NSs active at GABA_A_Rs [85]. These findings open intriguing possibilities, as molecules and enzymes that regulate plant steroid biosynthesis could interact through the diet with the human neurosteroid metabolism.

Adding another layer of potential interaction amongst botanicals, NSs, and GABA_A_Rs, recent computer modelling studies suggest that sesquiterpenes and sesquiterpenoids, present in plants such as hops and chamomile, enhance GABA_A_R function via the NS binding site [86]. Thus, NS-like compounds are also being explored as new potential components with biological activity in herbal alcohol alternatives.

## 8. Other Possible Molecular GABA Targets

### GABA_B_ and GHB Receptors

The use of phenibut by Kin raises the question of targeting GABA_B_R to mimic the effects of alcohol [87]. The old anti-spasticity agent baclofen is the classic GABA_B_R agonist and in humans is sedating, and to a limited extent similar in psychological effects to alcohol in normal healthy volunteers, though much more sedating [88]. This has led to its being used as a treatment for alcohol dependence in some countries, most notably France [89]. In this case it is used in very high doses to reduce alcohol use and craving, where it can be seen as a substitute for alcohol [90].

Sodium oxybate (trade name ALCOVER) is an agonist at the GHBR but also probably acts via the GABA_B_Rs too. It is licensed as a treatment for alcohol dependence in Italy and Austria and is under consideration across Europe. As with baclofen, it reduces craving for but also blocks withdrawal from alcohol [91] in people who are not alcohol-dependent and so cross-tolerant to it. Sodium oxybate is very sedating and so is also licensed to promote deep sleep in patients with narcolepsy. Under the slang name GHB it is quite widely used as a recreational sedative. Its abuse has been associated with deaths from overdose, and tolerance with severe withdrawal is well-recognised.

Taken together, these data suggest that directly activating GABA_B_R or GHBR is not a viable approach to developing a safe and well-tolerated alcohol alternative. However, the recent development of GABA_B_R PAMs, e.g., ADX71441 and ODM-106, which exhibit an improved, favourable behavioural profile in animal models, suggests that in common with GABA_A_R PAMs, GABA_B_R PAMs may offer a plausible safe alternative to alcohol (for an updated discussion see [91]).

## 9. From Bench to Bar: What Are the Regulatory and Other Challenges?

The vision of functional alcohol alternatives as introduced by the UK government Foresight report of 2005 [6] is near to being realised. Several botanical drinks are currently marketed, with customer reports of positive effects. At least one, Sentia Red, contains food and herbal products that allow it to meet the UK and European food safety standards as a food supplement. Others contain some ingredients, e.g., lion’s mane mushrooms, which have not yet passed this test but may yet become approved [92].

The challenge for a synthetic ingredient is to become approved as a food similar to alcohol, or at least as a food supplement such as other functional products, e.g., 5-HTP. In the USA, one pathway is via the GRAS (generally recognized as safe) route. Here an FDA-approved set of toxicology tests are performed, and the results are then reviewed by an expert panel who decide if the product is safe or not. In Europe, the European Food Safety Authority (EFSA) has the same role as the FDA but does not have a GRAS-equivalent route. The UK has not as yet announced if it will change its procedures for safety testing of potential novel foods post-Brexit or stay with the European ones.

Another important issue is how and where to make alcohol alternatives available for sale. Obviously, they should be in bars, pubs, and restaurants, though current UK regulations do not insist on non-alcohol drinks being made available. However, what about in shops and supermarkets? Should they be in the alcohol drinks aisles or in the soft drinks sections? We argue that to maximise their potential to reduce alcohol intake and hence harms they should be sold alongside alcoholic beverages, in the same way as low-alcohol drinks are. This would make them a more obvious choice for those people looking to reduce their alcohol intake.

An important consideration here is that these new alcohol alternatives are not designed to help dependent drinkers stop their use of alcohol. Rather, the field sees them as providing choice for drinkers, especially the young. Given the median age for alcohol dependence in the UK is 15 years and in most countries dependence similarly starts 3 years before the legal drinking age, any reduction in consumption of alcohol in the young is likely to have enduring health impacts. In this regard, the recent introduction of minimum unit pricing in Scotland was estimated to have very significant health impacts despite only producing a 10% reduction in consumption. Should functional alcohol alternatives achieve a similar impact on alcohol consumption, then similar health benefits can be predicted.

## References

[1] Nutt D.J., King L.A., Phillips L.D., Independent Scientific Committee on Drugs (2010). Drug harms in the UK: A multicriteria decision analysis. Lancet.

[2] van Amsterdam J., Nutt D., Phillips L., van den Brink W. (2015). European rating of drug harms. J. Psychopharmacol..

[3] Bonomo Y., Norman A., Biondo S., Bruno R., Daglish M., Dawe S., Egerton-Warburton D., Karro J., Kim C., Lenton S. (2019). The Australian drug harms ranking study. J. Psychopharmacol..

[4] Nutt D.J., Gual A., Anderson P., Rehm J. (2019). Why Less Is Always More in the Treatment of Alcohol Use Disorders. JAMA Psychiatry.

[5] Nutt D.J. (2006). Alcohol alternatives—A goal for psychopharmacology?. J. Psychopharmacol..

[6] Foresight R. How Future Scientific Advances May Affect Our Understanding of Addiction and Drug Use. https://www.gov.uk/government/publications/drugs-futures-2025.

[7] Nutt D., Hayes A., Fonville L., Zafar R., Palmer E.O.C., Paterson L., Lingford-Hughes A. (2021). Alcohol and the Brain. Nutrients.

[8] Abrahao K.P., Salinas A.G., Lovinger D.M. (2017). Alcohol and the Brain: Neuronal Molecular Targets, Synapses, and Circuits. Neuron.

[9] Roerecke M. (2021). Alcohol’s Impact on the Cardiovascular System. Nutrients.

[10] Pohl K., Moodley P., Dhanda A.D. (2021). Alcohol’s Impact on the Gut and Liver. Nutrients.

[11] Rumgay H., Murphy N., Ferrari P., Soerjomataram I. (2021). Alcohol and Cancer: Epidemiology and Biological Mechanisms. Nutrients.

[12] Slingerland E. (2021). Drunk: How We Sipped, Danced, and Stumbled Our Way to Civilization.

[13] Lembke A. (2021). Dopamine Nation: Finding Balance in the Age of Indulgence..

[14] Hoffman P.L., Rabe C.S., Grant K.A., Valverius P., Hudspith M., Tabakoff B. (1990). Ethanol and the NMDA receptor. Alcohol.

[15] Kobayashi N.H.C., Farias S.V., Luz D.A., Machado-Ferraro K.M., Conceicao B.C.D., Silveira C., Fernandes L.M.P., Cartagenes S.C., Ferreira V.M.M., Fontes-Junior E.A. (2022). Ketamine plus Alcohol: What We Know and What We Can Expect about This. Int. J. Mol. Sci..

[16] Lovinger D.M. (1999). 5-HT3 receptors and the neural actions of alcohols: An increasingly exciting topic. Neurochem. Int..

[17] Koob G.F., Buck C.L., Cohen A., Edwards S., Park P.E., Schlosburg J.E., Schmeichel B., Vendruscolo L.F., Wade C.L., Whitfield T.W. (2014). Addiction as a stress surfeit disorder. Neuropharmacology.

[18] Crews F.T., Lawrimore C.J., Walter T.J., Coleman L.G. (2017). The role of neuroimmune signaling in alcoholism. Neuropharmacology.

[19] Quillin S.J., Tran P., Prindle A. (2021). Potential Roles for Gamma-Aminobutyric Acid Signaling in Bacterial Communities. Bioelectricity.

[20] Cryan J.F., O’Riordan K.J., Cowan C.S.M., Sandhu K.V., Bastiaanssen T.F.S., Boehme M., Codagnone M.G., Cussotto S., Fulling C., Golubeva A.V. (2019). The Microbiota-Gut-Brain Axis. Physiol. Rev..

[21] Strandwitz P., Kim K.H., Terekhova D., Liu J.K., Sharma A., Levering J., McDonald D., Dietrich D., Ramadhar T.R., Lekbua A. (2019). GABA-modulating bacteria of the human gut microbiota. Nat. Microbiol..

[22] Barrett E., Ross R.P., O’Toole P.W., Fitzgerald G.F., Stanton C. (2012). gamma-Aminobutyric acid production by culturable bacteria from the human intestine. J. Appl. Microbiol..

[23] Otaru N., Ye K., Mujezinovic D., Berchtold L., Constancias F., Cornejo F.A., Krzystek A., de Wouters T., Braegger C., Lacroix C. (2021). GABA Production by Human Intestinal Bacteroides spp.: Prevalence, Regulation, and Role in Acid Stress Tolerance. Front. Microbiol..

[24] Tillisch K., Mayer E.A., Gupta A., Gill Z., Brazeilles R., Le Neve B., van Hylckama Vlieg J.E.T., Guyonnet D., Derrien M., Labus J.S. (2017). Brain Structure and Response to Emotional Stimuli as Related to Gut Microbial Profiles in Healthy Women. Psychosom. Med..

[25] Belelli D., Hales T.G., Lambert J.J., Luscher B., Olsen R., Peters J.A., Rudolph U., Sieghart W. (2021). GABAA receptors in GtoPdb v.2021.3. IUPHAR/BPS Guide Pharmacol. CITE.

[26] Alexander S.P., Christopoulos A., Davenport A.P., Kelly E., Mathie A., Peters J.A., Veale E.L., Armstrong J.F., Faccenda E., Harding S.D. (2021). THE CONCISE GUIDE TO PHARMACOLOGY 2021/22: G protein-coupled receptors. Br. J. Pharmacol..

[27] Bassetti D. (2022). Keeping the Balance: GABAB Receptors in the Developing Brain and Beyond. Brain Sci..

[28] Wisden W., Laurie D.J., Monyer H., Seeburg P.H. (1992). The distribution of 13 GABAA receptor subunit mRNAs in the rat brain. I. Telencephalon, diencephalon, mesencephalon. J. Neurosci..

[29] Maramai S., Benchekroun M., Ward S.E., Atack J.R. (2020). Subtype Selective gamma-Aminobutyric Acid Type A Receptor (GABAAR) Modulators Acting at the Benzodiazepine Binding Site: An Update. J. Med. Chem..

[30] Jackson A., Stephens D.N., Duka T. (2003). Lorazepam substitutes for the alcohol stimulus in social drinkers. Psychopharmacology.

[31] Belelli D., Lambert J.J. (2005). Neurosteroids: Endogenous regulators of the GABA(A) receptor. Nat. Rev. Neurosci..

[32] Hosie A.M., Wilkins M.E., da Silva H.M., Smart T.G. (2006). Endogenous neurosteroids regulate GABAA receptors through two discrete transmembrane sites. Nature.

[33] Laverty D., Thomas P., Field M., Andersen O.J., Gold M.G., Biggin P.C., Gielen M., Smart T.G. (2017). Crystal structures of a GABAA-receptor chimera reveal new endogenous neurosteroid-binding sites. Nat. Struct. Mol. Biol..

[34] Miller P.S., Scott S., Masiulis S., De Colibus L., Pardon E., Steyaert J., Aricescu A.R. (2017). Structural basis for GABAA receptor potentiation by neurosteroids. Nat. Struct. Mol. Biol..

[35] Chen Q., Wells M.M., Arjunan P., Tillman T.S., Cohen A.E., Xu Y., Tang P. (2018). Structural basis of neurosteroid anesthetic action on GABAA receptors. Nat. Commun..

[36] Carter R.B., Wood P.L., Wieland S., Hawkinson J.E., Belelli D., Lambert J.J., White H.S., Wolf H.H., Mirsadeghi S., Tahir S.H. (1997). Characterization of the anticonvulsant properties of ganaxolone (CCD 1042; 3alpha-hydroxy-3beta-methyl-5alpha-pregnan-20-one), a selective, high-affinity, steroid modulator of the gamma-aminobutyric acid(A) receptor. J. Pharmacol. Exp. Ther..

[37] Knight E.M.P., Amin S., Bahi-Buisson N., Benke T.A., Cross J.H., Demarest S.T., Olson H.E., Specchio N., Fleming T.R., Aimetti A.A. (2022). Safety and efficacy of ganaxolone in patients with CDKL5 deficiency disorder: Results from the double-blind phase of a randomised, placebo-controlled, phase 3 trial. Lancet Neurol..

[38] Belelli D., Hogenkamp D., Gee K.W., Lambert J.J. (2020). Realising the therapeutic potential of neuroactive steroid modulators of the GABAA receptor. Neurobiol. Stress.

[39] Althaus A.L., Ackley M.A., Belfort G.M., Gee S.M., Dai J., Nguyen D.P., Kazdoba T.M., Modgil A., Davies P.A., Moss S.J. (2020). Preclinical characterization of zuranolone (SAGE-217), a selective neuroactive steroid GABAA receptor positive allosteric modulator. Neuropharmacology.

[40] Deligiannidis K.M., Meltzer-Brody S., Gunduz-Bruce H., Doherty J., Jonas J., Li S., Sankoh A.J., Silber C., Campbell A.D., Werneburg B. (2021). Effect of Zuranolone vs Placebo in Postpartum Depression: A Randomized Clinical Trial. JAMA Psychiatry.

[41] Gunduz-Bruce H., Takahashi K., Huang M.Y. (2022). Development of neuroactive steroids for the treatment of postpartum depression. J. Neuroendocrinol..

[42] Bowen C.A., Purdy R.H., Grant K.A. (1999). Ethanol-like discriminative stimulus effects of endogenous neuroactive steroids: Effect of ethanol training dose and dosing procedure. J. Pharmacol. Exp. Ther..

[43] Allen D.C., Ford M.M., Grant K.A. (2018). Cross-Species Translational Findings in the Discriminative Stimulus Effects of Ethanol. Curr. Top. Behav. Neurosci..

[44] Helms C.M., McCracken A.D., Heichman S.L., Moschak T.M. (2013). Ovarian hormones and the heterogeneous receptor mechanisms mediating the discriminative stimulus effects of ethanol in female rats. Behav. Pharmacol..

[45] Finn D.A., Beadles-Bohling A.S., Beckley E.H., Ford M.M., Gililland K.R., Gorin-Meyer R.E., Wiren K.M. (2006). A new look at the 5alpha-reductase inhibitor finasteride. CNS Drug Rev..

[46] Khisti R.T., VanDoren M.J., O’Buckley T., Morrow A.L. (2003). Neuroactive steroid 3 alpha-hydroxy-5 alpha-pregnan-20-one modulates ethanol-induced loss of righting reflex in rats. Brain Res..

[47] Hirani K., Sharma A.N., Jain N.S., Ugale R.R., Chopde C.T. (2005). Evaluation of GABAergic neuroactive steroid 3alpha-hydroxy-5alpha-pregnane-20-one as a neurobiological substrate for the anti-anxiety effect of ethanol in rats. Psychopharmacology.

[48] VanDoren M.J., Matthews D.B., Janis G.C., Grobin A.C., Devaud L.L., Morrow A.L. (2000). Neuroactive steroid 3alpha-hydroxy-5alpha-pregnan-20-one modulates electrophysiological and behavioral actions of ethanol. J. Neurosci..

[49] Hirani K., Khisti R.T., Chopde C.T. (2002). Behavioral action of ethanol in Porsolt’s forced swim test: Modulation by 3 alpha-hydroxy-5 alpha-pregnan-20-one. Neuropharmacology.

[50] Torres J.M., Ortega E. (2003). Alcohol intoxication increases allopregnanolone levels in female adolescent humans. Neuropsychopharmacology.

[51] Torres J.M., Ortega E. (2004). Alcohol intoxication increases allopregnanolone levels in male adolescent humans. Psychopharmacology.

[52] Morrow A.L., Boero G., Porcu P. (2020). A Rationale for Allopregnanolone Treatment of Alcohol Use Disorders: Basic and Clinical Studies. Alcohol. Clin. Exp. Res..

[53] Durkin E.J., Muessig L., Herlt T., Lumb M.J., Patel R., Thomas P., Bright D.P., Jurd R., Moss S.J., Dickenson A.H. (2018). Brain neurosteroids are natural anxiolytics targeting α2 subunit γ-aminobutyric acid type-A receptors. BioRXiv.

[54] Belelli D., Phillips G.D., Atack J.R., Lambert J.J. (2022). Relating neurosteroid modulation of inhibitory neurotransmission to behaviour. J. Neuroendocrinol..

[55] Zolpidem. https://www.drugs.com/zolpidem.html#.

[56] Zoplicone. https://www.drugs.com/zopiclone.html.

[57] Sikdar S. (1998). Physical dependence on zopiclone. Prescribing this drug to addicts may give rise to iatrogenic drug misuse. BMJ.

[58] June H.L., Foster K.L., McKay P.F., Seyoum R., Woods J.E., Harvey S.C., Eiler W.J., Grey C., Carroll M.R., McCane S. (2003). The reinforcing properties of alcohol are mediated by GABA(A1) receptors in the ventral pallidum. Neuropsychopharmacology.

[59] Atack J.R. (2011). GABAA receptor subtype-selective modulators. I. alpha2/alpha3-selective agonists as non-sedating anxiolytics. Curr. Top. Med. Chem..

[60] AZD7325 Clinical Trial. https://www.clinicaltrials.gov/ct2/results?term=AZD7325&Search=Sear.

[61] June H.L., Harvey S.C., Foster K.L., McKay P.F., Cummings R., Garcia M., Mason D., Grey C., McCane S., Williams L.S. (2001). GABA(A) receptors containing (alpha)5 subunits in the CA1 and CA3 hippocampal fields regulate ethanol-motivated behaviors: An extended ethanol reward circuitry. J. Neurosci..

[62] Zhu S., Noviello C.M., Teng J., Walsh R.M., Kim J.J., Hibbs R.E. (2018). Structure of a human synaptic GABAA receptor. Nature.

[63] Masiulis S., Desai R., Uchanski T., Serna Martin I., Laverty D., Karia D., Malinauskas T., Zivanov J., Pardon E., Kotecha A. (2019). GABAA receptor signalling mechanisms revealed by structural pharmacology. Nature.

[64] Kim J.J., Gharpure A., Teng J., Zhuang Y., Howard R.J., Zhu S., Noviello C.M., Walsh R.M., Lindahl E., Hibbs R.E. (2020). Shared structural mechanisms of general anaesthetics and benzodiazepines. Nature.

[65] Sauguet L., Shahsavar A., Poitevin F., Huon C., Menny A., Nemecz A., Haouz A., Changeux J.P., Corringer P.J., Delarue M. (2014). Crystal structures of a pentameric ligand-gated ion channel provide a mechanism for activation. Proc. Natl. Acad. Sci. USA.

[66] Forstera B., Castro P.A., Moraga-Cid G., Aguayo L.G. (2016). Potentiation of Gamma Aminobutyric Acid Receptors (GABAAR) by Ethanol: How Are Inhibitory Receptors Affected?. Front. Cell. Neurosci..

[67] Greenblatt D.J., Shader R.I., Divoll M., Harmatz J.S. (1981). Benzodiazepines: A summary of pharmacokinetic properties. Br. J. Clin. Pharmacol..

[68] Kinnersley A.M., Turano F.J. (2000). Gamma aminobutyric acid (GABA) and plant responses to stress. Crit. Rev. Plant Sci..

[69] Li L., Dou N., Zhang H., Wu C.X. (2021). The versatile GABA in plants. Plant Signal. Behav..

[70] Cullinan W.E., Ziegler D.R., Herman J.P. (2008). Functional role of local GABAergic influences on the HPA axis. Brain Struct. Funct..

[71] Cicek S.S. (2018). Structure-Dependent Activity of Natural GABA(A) Receptor Modulators. Molecules.

[72] Atal C.K., Dubey R.K., Singh J. (1985). Biochemical Basis of Enhanced Drug Bioavailability by Piperine—Evidence That Piperine Is a Potent Inhibitor of Drug-Metabolism. J. Pharmacol. Exp. Ther..

[73] Zhang Q.L., Fu B.M.M., Zhang Z.J. (2017). Borneol, a novel agent that improves central nervous system drug delivery by enhancing blood-brain barrier permeability. Drug Deliv..

[74] Sinyard A. Sentia: Is this Alcohol-Free Spirit that Makes You “Tipsy” the Future of Drinking?. https://www.stylist.co.uk/health/alcohol-free-spirit-tipsy-sentia/574441.

[75] Schuster-Bruce C. I Tried an Alcohol-Free, No-Hangover Drink Made by a Top Professor that Claims to Make You as Relaxed as Alcohol Does. It Hits the Spot—but Make Sure You Read the Label. https://www.businessinsider.com/i-tried-alcohol-free-drink-professor-relaxed-alcohol-no-hangover-2021-3?r=US&IR=T.

[76] Derrick F. This Alcohol-Free Spirit Promises to Give You All the Good Bits of Booze—And None of the Bad. https://metro.co.uk/2021/01/22/alcohol-free-spirit-sentia-promises-good-effects-of-booze-without-a-hangover-13947226/.

[77] Kin Euphorics. https://www.kineuphorics.com/.

[78] Lapin I. (2001). Phenibut (beta-phenyl-GABA): A tranquilizer and nootropic drug. CNS Drug Rev..

[79] U.S. Food and Drug Administration (FDA) FDA Acts on Dietary Supplements Containing DMHA and Phenibut. https://www.fda.gov/food/cfsan-constituent-updates/fda-acts-dietary-supplements-containing-dmha-and-phenibut.

[80] Boonstra E., de Kleijn R., Colzato L.S., Alkemade A., Forstmann B.U., Nieuwenhuis S. (2015). Neurotransmitters as food supplements: The effects of GABA on brain and behavior. Front. Psychol..

[81] Three Spirit. https://threespiritdrinks.com/.

[82] Grundmann O., Wang J., McGregor G.P., Butterweck V. (2008). Anxiolytic Activity of a Phytochemically Characterized Passiflora incarnata Extract is Mediated via the GABAergic System. Planta Med..

[83] Awad R., Muhammad A., Durst T., Trudeau V.L., Arnason J.T. (2009). Bioassay-guided Fractionation of Lemon Balm (*Melissa officinalis* L.) using an In Vitro Measure of GABA Transaminase Activity. Phytother. Res..

[84] Rosati F., Danza G., Guarna A., Cini N., Racchi M.L., Serio M. (2003). New evidence of similarity between human and plant steroid metabolism: 5alpha-reductase activity in Solanum malacoxylon. Endocrinology.

[85] Rosati F., Bardazzi I., De Blasi P., Simi L., Scarpi D., Guarna A., Serio M., Racchi M.L., Danza G. (2005). 5alpha-Reductase activity in Lycopersicon esculentum: Cloning and functional characterization of LeDET2 and evidence of the presence of two isoenzymes. J. Steroid Biochem. Mol. Biol..

[86] Janzen D., Slavik B., Zehe M., Sotriffer C., Loos H.M., Buettner A., Villmann C. (2021). Sesquiterpenes and sesquiterpenoids harbor modulatory allosteric potential and affect inhibitory GABAA receptor function in vitro. J. Neurochem..

[87] Tyacke R.J., Lingford-Hughes A., Reed L.J., Nutt D.J. (2010). GABAB receptors in addiction and its treatment. Adv. Pharmacol..

[88] Durant C.F., Paterson L.M., Turton S., Wilson S.J., Myers J.F.M., Muthukumaraswamy S., Venkataraman A., Mick I., Paterson S., Jones T. (2018). Using Baclofen to Explore GABA-B Receptor Function in Alcohol Dependence: Insights From Pharmacokinetic and Pharmacodynamic Measures. Front. Psychiatry.

[89] Rolland B., Simon N., Franchitto N., Aubin H.J. (2020). France Grants an Approval to Baclofen for Alcohol Dependence. Alcohol Alcohol..

[90] Chick J., Nutt D.J. (2012). Substitution therapy for alcoholism: Time for a reappraisal?. J. Psychopharmacol..

[91] Augier E. (2021). Recent Advances in the Potential of Positive Allosteric Modulators of the GABAB Receptor to Treat Alcohol Use Disorder. Alcohol Alcohol..

[92] EU Novel Food Catalogue 2022. https://webgate.ec.europa.eu/fip/novel_food_catalogue/#.

